# Social and ethical impact of emotional AI advancement: the rise of pseudo-intimacy relationships and challenges in human interactions

**DOI:** 10.3389/fpsyg.2024.1410462

**Published:** 2024-11-05

**Authors:** Jie Wu

**Affiliations:** School of Journalism and Communication, Renmin University of China, Beijing, China

**Keywords:** emotion, algorithm, user-platform relationship, pseudo-intimacy relationship, paradigm for human interaction

## 1 Introduction

Algorithmic platforms, as a form of intelligent digital infrastructure underpinned by algorithms, have fundamentally transformed how individuals connect and interact with one another (Yin and Lin, 2023). The integration of emotional intelligence into these algorithms further deepens the relational connections between users and platforms. By enhancing the algorithm's affective capabilities—such as emotion perception and feedback (Wu et al., 2022; Bie and Zeng, 2024; Peng, 2024)—the attributes of user-platform relationships undergo qualitative changes within the affective dimension. Consequently, the model of human-computer interaction (HCI) evolves toward a trend of humanization, as articulated by Paul (2017). To some extent, this not only realizes the technical possibility of platform personification but also addresses modern individuals' emotional needs within Cyborg space. This evolution fosters and sustains potential connections in the emotional dimension between users and platforms (Lai, 2023; Hong and Huang, 2024). As a result, emotions have emerged as a significant focus in research concerning interactions between users and algorithmic platforms.

Going back in history, Marvin Minsky, regarded as the father of artificial intelligence (AI), proposed the groundbreaking idea that “AI should possess emotions” as early as 1985 (Marvin, 2006). Subsequently, Rosalind Picard, in her seminal work *Affective Computing*, further elucidated the technical possibilities of endowing computers with emotional capabilities (Rosalind, 1997). Numerous studies in media psychology have demonstrated that individuals often mindlessly equate virtual media featuring anthropomorphic cues with real-life experiences (Reeves and Nass, 1996), leading to para-social interactions and relationships with these entities (Rubin et al., 1985; Bickmore and Picard, 2005). However, at that time, this conclusion was constrained by limitations within computer science and artificial intelligence fields regarding affective computing technology. Consequently, it primarily existed at the level of academic experiments and discussions without widespread empirical evidence in real-world contexts. In recent years, however, the rapid advancement and extensive application of emotional AI have transcended these academic boundaries. This evolution has led to para-social relationships becoming increasingly normalized within society—an occurrence that is now garnering significant attention from a diverse array of scholars across the humanities and social sciences.

Unlike scholars in the field of affective computing, who primarily focus on experimental research involving HCI, those in the humanities and social sciences tend to emphasize the exploration of the social and ethical risks associated with these technologies from philosophical and sociological perspectives. For instance, Marx famously asserted that the essence of humanity is “the sum of all social relations” (Marx Engels, 2009). Consequently, understanding what it means to be human necessitates an examination of the relationships between individuals and others. Building upon this foundation, some scholars have suggested that within the current dynamics of user-platform interactions, integrating emotional intelligence into the development of human-like AI (such as algorithms) may give rise to a phenomenon termed “human alienation.” This occurs when AI—a product of human creation—poses a threat to the evolution of human subjectivity across three dimensions: communication, cognition, and labor (Xie and Liu, 2023). Thus, they call for society at large to recognize the developmental limits of AI and advocate for creating controllable, safe, and reliable AI systems while promoting a collaborative evolution between human-machine societies and general AI (Huang and Lv, 2023).

Against this backdrop, I found that, despite the existence of numerous studies exploring the emotional interactions between humans and computers (Rosalind, 1997; Reeves and Nass, 1996; McStay, 2018; Marcos-Pablos and García-Peñalvo, 2022; Peng, 2024; Lai, 2023; Gan and Wang, 2024; Zhao and Li, 2023), as well as the ethical issues arising from the development of emotional AI (Gossett, 2023; Tretter, 2024; Nyholm and Frank, 2019; Xiao and Zhang, 2024; Yin and Liu, 2021; Zhang, 2024), so far, there has been no research that approached the issue from a theoretical and speculative standpoint, focusing on the definition of the dynamic emotional interaction relationship between users and AI platforms with the development of emotional AI, and further explores how it impacts human interaction paradigms. Specifically, there is a lack of theoretical discussion on the profound changes in existing societal paradigms brought about by the advancement of emotional AI.

My goal is to fill this gap and to argue that, when algorithms integrate emotional intelligence, a new type of relationship—pseudo-intimacy—emerges between users and platforms, serving as a new paradigm of human interaction that coexists with face-to-face relationships in the real world. In this pseudo-intimacy relationship, on the one hand, users and platforms achieve instantaneous emotional interaction, partially satisfying the human's desire for intimacy. However, it is also restricted by the limited development of emotional AI and human irrationality, making the human social environment more full of contradictions and tension. Consequently, the advancement of emotional AI should not only focus on technological innovation and subjective human experiences but also fully consider its impact on human social interaction paradigms. Yet, if appropriate measures are taken to address these ethical risks, I argue, nothing can fundamentally stand in the way of the progress of emotional AI.

To elaborate on my thesis, I will first focus on how the pseudo-intimacy relationship emerges and develops, and provide a realistic explanation of its definition. Then, I will conduct a summary discussion on the relevant ethical risks, and express my attitude and recommendations toward the future development of emotional AI.

## 2 The pseudo-intimacy relationship of user-platform becomes a new paradigm for human interaction

The human-computer society could not exist without emotions assuming the role of the glue (Gan and Wang, 2024). However, although emotions in HCI have received attention from scholars of affective computing, such as Rosalind Pickard, since the end of the last century, and “para-social relationship” has been discussed in media studies for decades, yet emotions have been marginalized in the study of user-platform relationships in the field of sociology for a long time. This is mainly due to the stereotype of “emotion-rationality” dichotomy among some scholars (Yuan, 2021). In recent years, research within social robotics has made significant strides in enhancing robots' emotional capabilities to improve their capacity for empathy and social engagement with humans (Marcos-Pablos and García-Peñalvo, 2022). Sociological theorists are increasingly recognizing that, along with the algorithmic platform's anthropomorphic development (Wu et al., 2022; Zhao and Li, 2023), the most distinct boundary between HCI and interpersonal social interaction—the authenticity of the interaction object (Giles, 2002)—has been broken. The user-platform relationship has beyond the “para-social relationship” defined by HCI scholars, resulting in a “pseudo-intimacy relationship” between humans and humanlike entities. This is evident in current HCI, where users anthropomorphize and idealize computers based on their emotional intelligence, forming social relationships that are more satisfying than face-to-face ones.

### 2.1 The user-platform emotional relationship is thoroughly elucidated in the context of immediate interaction, and partially satisfying the human need for intimacy

Anthropocentrism posits that humans have an inherent tendency to anthropomorphize non-human entities, driven by a desire to engage and connect with society (Epley et al., 2007). In *Alone Together*, Sherry Turkle, a professor of sociology at the Massachusetts Institute of Technology (MIT), examined the psychological phenomenon whereby individuals forge intimate connections with computers. She argues that humans can develop emotional relationships with computers, even may regard them as significant others akin to family and friends (Sherry, 2014). This human-computer relationship established on this premise—particularly in the context of social media—mimics emotional bonds found among humans. However, it lacks the depth and complexity characteristic of genuine human interactions, which is somewhat constrained by the technological advancements available at that time. Since then, scholars have increasingly suggested that individuals may integrate computers into their interpersonal networks and become emotionally reliant on their presence (Thomas and Julia, 2018; Wang, 2023; Wang et al., 2024; Gan and Wang, 2024).

With the rapid advancement of AI's emotional capabilities and the widespread adoption of intelligent algorithmic platforms, this perspective is increasingly validated. Algorithmic technologies endowed with emotional intelligence facilitate instantaneous bidirectional interactions between users and platforms within the realm of emotional communication (Ke and Song, 2021; Hong and Huang, 2024). Based on the emotional purpose of human communication, this paper characterizes it as “pseudo-intimacy relationship.” In this relationship, due to the lack of non-verbal social cues in face-to-face interactions, instant emotional interactions between users and platforms mediated by affective AI may lead users to overinterpret limited information (Walther et al., 2015), thereby leading to an unhealthy development of the relationship between the two.

In terms of emotional interaction, the enhancement of algorithmic emotional intelligence not only made algorithmic platforms novel objects of human interaction but also awakened and partially satisfied the latent human need for intimacy. Some researchers have noted that this enhancement facilitates the mobilization of human emotions for immediate user-platform emotional interactions (Bie, 2023). With emotional intelligence, users display strong conscious or unconscious emotions during interactions (Nagy and Neff, 2015), and continuously motivating themselves to engage while also eliciting immediate emotional feedback from the platform, thereby accelerating the emotional flow between them.

In addition, from the point of view of the “mirror me” theory put forward by American sociologist Charles Horton Cooley, the essence of user-platform emotional interaction is an extension of human emotion projection and the construction of the ideal self in social interaction (Gan and Wang, 2024). In the dynamic interplay of human emotional projection and computer affective computing, a recursive effect akin to an “infinite mirror” emerges between the two entities (Panaite and Bogdanffy, 2019), wherein emotions are continuously iterated and refined. This process fosters the evolution of user-platform communication forms and experiences, with pseudo-intimacy becoming a defining characteristic of the user-platform relationship. This further deepens the emotional exchange between users and platforms, potentially elevating it to a cultural level and generating consensus on granting platforms the status of “interaction subjects” in society, and even envisioning a future where user-platform emotional exchanges are equalized.

However, it must be pointed out that, in contrast to the technological object essence of emotional AI, only human beings are truly emotional animals. Emotions, as a reflection of collective human intentions, are expressed through and rationalize human behavior (Swallow, 2009). As a result, regarding the evolution of user-platform relationship attributes, emotional intelligence in AI systems is an external factor, while the human need for intimacy is the initial driving force that promotes pseudo-intimacy relationship to be a new paradigm of human interaction.

### 2.2 User-platform emotional interactions have become more real and tangible, while the human social environment characterized by heightened contradictions and tensions

From the perspective of social relationships, before algorithmic emotional capabilities were developed, the user-platform relationship was fundamentally an HCI. Even with emotional undertones, it remains a one-sided contribution from users, who receive no emotional response from platforms and only feedback on usage and satisfaction—referred to as “user stickiness” (Periaiya and Nandukrishna, 2024). In contemporary times, with further developments in emotional AI technology, algorithmic platforms are now endowed with emotional capabilities. The anthropomorphic affective attributes within the user-platform relationship have become more pronounced in communicative contexts (Zhejiang Lab and Deloitte, 2023). This evolution has introduced a degree of warmth into these interactions, leading to the emergence and implementation of conversational and companionable AI.

However, akin to two sides of the same coin, the development of emotional intelligence in AI systems has also introduced a range of associated risks and sparked extensive discussions regarding their ethical implications within studies (McStay, 2018; Greene, 2020; Gremsl and Hödl, 2022; Gossett, 2023; Tretter, 2024). These discussions highlight the potential benefits of emotionally capable AI systems while simultaneously addressing the challenges posed by the technological uncontrollability of AI companions and human irrationality in emotional ineractions with intelligent systems (Yang and Wu, 2024; Chen and Tang, 2024). Scholars contend that as long as emotional AI technologies can influence human emotions, they possess the potential to serve as instruments of emotional deception (Bertolini and Arian, 2020). In light of these concerns, many researchers advocate for implementing protective measures across various fields such as education, healthcare, and justice to regulate AI systems capable of interpreting and responding to human emotions while preventing their irrational use (McStay, 2020; Vagisha and Harendra, 2023; Crawford, 2021).

In the context of the user-platform relationship that this article examines, the advancement of emotional AI technology has also exacerbated ethical concerns related to private data security, algorithmic bias leading to discrimination, and information cocooning (Mei, 2024; Yan et al., 2024). This is because, as the user-platform relationship becomes increasingly emotional, the relational attributes between a given platform and its different users may differ significantly. For platforms to sustain stable user-platform relationships, they must collect extensive data on users' emotional preferences and privacy information (Lu et al., 2022). However, current legislative frameworks regarding data protection in several countries with advanced platform technology development—such as China and the United States—remain incomplete. There are no uniform norms or standards governing how interest groups backing algorithmic platforms can protect or utilize such data. Grounded in media literacy, this situation has prompted a degree of self-reflexivity among users, leading them to develop concerns about risks associated with self-information security and emotional manipulation—commonly referred to as algorithmic anxiety (Cha et al., 2022).

In addition, from the perspective of the overall social environment, the current user-platform relationship can be characterized as a pseudo-intimacy relationship that does not exist in a seamless enclosure devoid or isolated space solely created by algorithmic platforms, AI, and other emotional agents. Instead, it coexists with genuine interpersonal socialization within real society, collectively forming a social environment rife with contradictions and tensions for individuals. Therefore, while the user-platform pseudo-intimacy relationship may enrich an individual's social life and alleviate loneliness to some extent (Yuan et al., 2024), it also impacts users' real-life interpersonal relationships. This influence can even adversely affect their social skills and attitudes, thereby hindering their understanding of interpersonal emotions and their significance, reducing opportunities for establishing more meaningful interactions (Sharkey and Sharkey, 2011; Nyholm and Frank, 2019). This negative impact arises because algorithmic platforms despite being programmed to understand and react to human emotions, it still lack the same innate capacity for empathy inherent in human beings (Morgante et al., 2024). Furthermore, the natural divide between humans and computers also leads users to perceive algorithmic platforms as a “quasi-other” (Mu and Wu, 2024). In this context, true reciprocal emotional communication between users and platforms has yet to be realized. The equalization of emotional communication between the two will continue to be a lengthy and challenging endeavor, hindered by both technical and ethical constraints.

## 3 Conclusions and future research

In summary, I argue that as emotional AI continues to develop, the user-algorithm platform relationship has shifted from a traditional HCI to a negotiating pseudo-intimacy between humans and humanlike entities. This is not only an imaginative, anthropomorphic, and social feature that emotional AI has bestowed upon HCI, but also an important supplement to human existing interaction paradigms. In addition, the emergence and development of pseudo-intimacy relationships partially satisfy human needs for intimacy in modern society; however, due to limitations about technological development and other factors, it is not entirely beneficial and raises ethical issues like privacy data security, increasing contradictions, and tension in the human social environment.

Therefore, we should agree that the advancement of emotional AI must focus not only on technological innovation but also on ethical constraints imposed by existing social norms, such as privacy security. Technological progress that violates ethical norms is always unacceptable. Also, in the face of the increasing emotional capabilities of algorithms, we should abandon the binary thinking of technology vs. humanity, rationality vs. emotion, and explore the harmonious coexistence of humanistic spirit and technological rationality in the future (Peng, 2021).

The discussion in this paper also has some limitations. Research on integrating emotional intelligence into algorithms, exploring the development of emotional functions within AI systems through methods such as human-computer experiments holds more significant practical application value. However, due to constraints related to genre, this paper primarily summarizes and examines these concepts at a theoretical level without engaging in large-scale experimental studies. Furthermore, as previously noted, the discourse surrounding ethical issues tacitly approve that we ought to allocate ethical responsibilities to AI technologies that are integrated with emotional intelligence. The reality, however, is that the questions of whether and how to continue to refine these technologies, whether and how to assign ethical responsibilities to them, and how humans should respond to the humanlike qualities of these technologies when interacting with them, are still under development and heated discussion. The answers need to be synthesized through data, theory, and other explorations by future researchers in computing science, humanities, social sciences, and other fields. Of course, it is also possible that there will not be a definite answer for a long period, which is also the charm of academic research.
